# Sports Diagnostics—Maximizing the Results or Preventing Injuries

**DOI:** 10.3390/ijerph20032470

**Published:** 2023-01-30

**Authors:** Krzysztof Mackala, Kamil Michalik, Hubert Makaruk

**Affiliations:** 1 Department of Track and Field, Wroclaw University of Health and Sport Sciences, 51-612 Wrocław, Poland; 2 Department of Human Motor Skills, Wroclaw University of Health and Sport Sciences, 51-612 Wrocław, Poland; 3 Faculty of Physical Education and Health, The Jozef Pilsudski University of Physical Education in Warsaw, 00-809 Biala Podlaska, Poland

## 1. Introduction

Sports diagnostics is a comprehensive scientific concept and comprises an aspect of training monitoring and/or sports medicine. In many cases, it is challenging to implement in the real world of sports, especially in the professional sphere [1,2]. It consists, among other aspects, of the medical control of both sick and healthy training competitors [3]; above all, this level of control allows fatigue to be managed and protects a competitor from the excessive risk of injury during intensive sports training [4].

One of the main goals of sports diagnosis is to maintain health, because it is on this basis that a regular training process is possible. Health—i.e., not just the ability to perform sports in general, but being healthy overall and free from injury after the end of a training session—is the smallest structure of the training process [5]. The maintenance of health allows one to break down the body’s barriers and perform more complex and extended efforts [6]. The right balance between training stimuli and rest allows one to adapt to new exercise conditions [7]. Due to this, it is possible to track the performance progress of an athlete. This applies to improving motor, technical, and even mental preparation in relation to the effort put in. We train to improve and achieve increasingly better sports-related results [8]. These elements, health and progress, are inextricably linked and result from one another; they penetrate each other. They show us what is meant by the term sports diagnostics.

The essence of sports diagnostics is the use of special procedures and tools necessary to control an athlete’s training process properly [9,10]. This applies to the assessment of the reaction of the athlete’s body—the system—to the training stimuli used in this process, i.e., the so-called training measures. This applies to both reactions/stimuli used in a single training unit of tasks (exercises), i.e., the current effect (acute responses), and the accumulation of many training sessions, e.g., carried out in a microcycle—and this will be a prolonged effect (chronic responses). Finally, long-term training effects are assessed, for example, in long mesocycles or macrocycles [11]. Here, we deal with the so-called cumulative effect. This shows us the state of a player’s actual training in a given training period, an annual cycle—that is, the athlete’s visible progress.

## 2. Selected Factors Proving the Importance of Sports Diagnostics

Due to the broad spectrum of knowledge regarding sports diagnostics, it is worth focusing on a few selected aspects within this issue [12]. Their common denominator is:

➢ Reference, i.e., the application of measurement or research methods which will determine the process of obtaining data, the way of interpreting it, and the possibility of their future modeling [6].
➢ Practical application: during training and in competition conditions, both in assessing the motor and technical preparation of a competitor in various sports disciplines as well as in determining the risk of injury or, in some cases, overtraining [8,9,10,11,12,13].
➢ The analyzed data concern accurate training loads obtained mainly in training and competition conditions—non-invasively, based on accurate, reliable, and above all, comprehensive measurements [10].
➢ Comprehensive measurements require the combination of methods which will allow for the assessment of the level of individual motor skills. This has to be carried out without disturbing motor structures, which fully provides for the actual implementation of technical tasks, and in the case of, e.g., games, technical and tactical tasks [13]. Such a comprehensive approach allows for a precise assessment—the actual conditions of applying training stimuli and their impact on an athlete’s body through mutual interaction between technique and physiological reactions and obtaining the optimal so-called readiness, i.e., reaching the state of the highest preparation [14,15]. To sum up, comprehensive measurements can reliably diagnose:
  - The body’s metabolic potential;
  - Motor potential;
  - Technical potential.

## 3. Areas of Sports Diagnostics

Sport diagnostics is closely related to training monitoring (external and internal loads). Thus, it may be an interesting complement and may contribute to knowledge regarding athletes and their training adaptations. Scientists should answer even the most difficult of questions from coaches. On the other hand, coaches should draw on their experiences and the newest knowledge. Below, some approaches which could be applied in the training process are presented.

### 3.1. Symmetry or Functional Asymmetry

This is related to the term lateralization, in other words, “laterality”—i.e., functional asymmetry of the right and left sides of the human body, which results from differences in the structure and functions of both cerebral hemispheres. Does one side have a functional advantage over the other? If so, is this advantage significant in achieving sports results, can it limit athletes, and can it even lead to functional disorders and exposure to injuries [16,17]?

### 3.2. Dynamic Module of Motor Skills

This is not only an assessment of the technique of performing a given movement structure but an assessment of the motor potential, mainly the level of strength and speed potential, and the result of their interaction—the so-called dynamic module. In addition, the peak power is needed to complete this structure [18,19].

### 3.3. Measurement of Muscle Strength

Muscular strength is overcoming or counteracting external resistance through athletic activity. It plays a dominant role in the motor preparation of an athlete [20]. It is based on the assessment of muscle strength moments of individual muscle groups (flexors and extensors) responsible for the function of individual joints [21]. The aim of this test is to determine the maximum value of muscle strength during movement in a given joint for both limbs—bilaterally—or for a single limb—unilaterally [22]. Different angular velocities enable the adjustment of the measurement to the implemented movement structure, a given sports discipline, and the subject’s disposition [23].

### 3.4. Peak Power Measurement

Why take such measurements? Does it seem reasonable to compare the size of the force measured in different conditions—especially those in static conditions (isometric) compared to dynamic conditions, but in a limited space (isokinetic measurement and the measurement of maximum force, e.g., a squat)—to actual dynamic conditions realized by performing vertical jumps (CMJ and DJ) [24] or horizontal standing long jumps [25] or alternating multiple jumps, analyzed based on one jump with both left and right limbs [26]? This comparison must be made in both the two-legged and one-legged measurements. Again, this is an issue related to lateralization or partialization. This power determines not only the quality of execution of a single movement structure, but above all, it is an exponent of the assessment of maintaining the peak power in the conditions of multiple repetitions of this structure [27].

### 3.5. Peak Power and Lactate Concentration

Few studies describe the relationship between the peak power and lactate levels in the blood, especially in the anaerobic lactic acid mechanism [28]. Lactic acid is produced in tissues during intense physical exercise when the metabolic requirements of the muscles increase and the supply of oxygen is insufficient. This, in turn, leads to an increase in the rate of glycolysis. Therefore, the rate of lactic acid production depends on the increasing intensity of exercise, which translates into an increase in the demand of working muscle cells for ATP [29]. Theoretically, an appropriate measurement of lactate concentration should indicate the level of the physical capacity of a given athlete and how a given training unit affects the body and also guide how training can be continued effectively [30].

### 3.6. Running Kinematics

To better understand the possibilities of using kinematic measurements in sports diagnostics, one should focus on the fundamental movement structure, which is movement with a change of body position, i.e., running, in various intensities and in different configurations. The analysis of the running technique is based on the analysis of a single running step, which ensures maximum effectiveness in its execution [31].

### 3.7. Measurement of Muscle and Tendon Stiffness and Flexibility

Another method is to assess speed and explosive power by measuring muscle stiffness and flexibility with a Myoton Pro device. Measurements concern:

Dynamic stiffness—a biomechanical property of the muscle characterized by resistance to contraction or opposition to an external force deforming its initial shape. In the case of abnormally high stiffness, a more significant effort of the agonist’s muscle is required to stretch the stiff antagonist, leading to an inefficient economy of movement [32]. In addition, increased muscle stiffness also creates unfavorable conditions for blood circulation and microcirculation, significantly reducing their ability to exercise.

Flexibility is a biomechanical property of a muscle which characterizes its ability to regain its original shape after the contraction or removal of an external force of deformation [33].

## 4. Conclusions

To sum up, it seems reasonable to ask another question: where does sports diagnostics come from, and above all, where should it go? The answer to this question, we may use a quote from former Lufthansa CEO Dr. Hans-Wilhem Muller-Wohlfarht. In his work, he was guided by the principle: “Theory is when everyone knows why, how to do it, but nothing works, practice is when everything works, but no one knows why”. By combining these two areas into one, not mutually exclusive relationship, we can precisely define the importance of diagnostics in sports and its place in the training process. Therefore, feedback from coaches and athletes whose needs result from the training process is essential for further theoretical research. It is possible to implement such feedback by modeling existing information or searching for entirely new information. Science can answer the questions that practice asks. Practice can use the achievements of research. In this context, the maximization of results and the prevention of injuries are among the most critical and mutual factors in a qualified sport.
